# Promising immunotherapeutic targets in lung cancer based on single-cell RNA sequencing

**DOI:** 10.3389/fimmu.2023.1148061

**Published:** 2023-04-28

**Authors:** Qianqian Xue, Wenbei Peng, Siyu Zhang, Xiaoshan Wei, Linlin Ye, Zihao Wang, Xuan Xiang, Pei Zhang, Qiong Zhou

**Affiliations:** Department of Respiratory and Critical Care Medicine, Union Hospital, Tongji Medical College, Huazhong University of Science and Technology, Wuhan, China

**Keywords:** lung cancer, tumor microenvironment, single-cell RNA sequencing, immune checkpoint blockade, immunotherapy

## Abstract

Immunotherapy has made great strides in the treatment of lung cancer, but a significant proportion of patients still do not respond to treatment. Therefore, the identification of novel targets is crucial to improving the response to immunotherapy. The tumor microenvironment (TME) is a complex niche composed of diverse pro-tumor molecules and cell populations, making the function and mechanism of a unique cell subset difficult to understand. However, the advent of single-cell RNA sequencing (scRNA-seq) technology has made it possible to identify cellular markers and understand their potential functions and mechanisms in the TME. In this review, we highlight recent advances emerging from scRNA-seq studies in lung cancer, with a particular focus on stromal cells. We elucidate the cellular developmental trajectory, phenotypic remodeling, and cell interactions during tumor progression. Our review proposes predictive biomarkers and novel targets for lung cancer immunotherapy based on cellular markers identified through scRNA-seq. The identification of novel targets could help improve the response to immunotherapy. The use of scRNA-seq technology could provide new strategies to understand the TME and develop personalized immunotherapy for lung cancer patients.

## 1 Introduction

Lung cancer is one of the most prevalent cancers with the highest mortality rates worldwide, with non-small-cell lung cancer (NSCLC) accounting for 85% of all lung cancer cases (1, 2). Recent advances in tumor immunotherapies aimed at reinvigorating tumor-infiltrating T cells have demonstrated clinical benefits in NSCLC, particularly with immune checkpoint blockade (ICB) treatment, including anti-programmed cell death protein-1 (PD-1)/PD-1 ligand-1 (PD-L1) and anti-cytotoxic T lymphocyte-associated antigen 4 (CTLA4) monoclonal antibody treatments. However, only a minority of patients achieve remarkable responses to ICB therapy due to intrinsic resistance to immunotherapy (3, 4). Extensive evidence has highlighted that dysfunctional tumor-killing T cells with high expression of immune inhibitory molecules, including PD-1, CTLA4, T cell membrane protein-3 (TIM-3), T cell immunoreceptor with immunoglobulin and ITIM domains (TIGIT), and lymphocyte-activation gene-3 (LAG-3), play crucial roles in non-response to ICB therapy within the TME (5). Furthermore, immunosuppressive cell subpopulations, such as regulatory T cells (Tregs), tumor-associated macrophages (TAMs), and cancer-associated fibroblasts (CAFs), impair effector T cell proliferation and cytotoxic responses, contributing to a dysfunctional T-cell state (6). Therefore, an in-depth understanding of the complex network of stromal cells within the TME is essential to addressing the non-responsiveness to immunotherapy in cancer patients.

Due to the heterogeneity and plasticity of stromal cells, identifying and defining distinct cell subsets is a significant challenge. With the booming development of high-dimensional profiling techniques, single-cell RNA sequencing (scRNA-seq) has emerged as a valuable tool to characterize the gene expression, compositional differences, and functional states of individual stromal cells. In addition, cell lineages, the dynamics of cell fate and differentiation dynamics, and crosstalk of stromal cell subsets in the TME can be revealed by analyzing of T-cell receptor (TCR) repertoires in scRNA-seq. These studies contribute to a better understanding of the potential roles of stromal cells in both tumor progression and resistance to ICB therapy (7, 8). In this review, we summarize recent advances in the TME landscape based on numerous scRNA-seq studies in lung cancer. In particular, we focused on stromal cells characterization, including their heterogeneity, dynamics, and potential functions in the TME. Moreover, we discuss the interactions between different subpopulations of stromal cells, as well as recent advances in immunotherapy strategies to identify effective predictive biomarkers and novel targets for lung cancer.

## 2 Phenotype remodeling of stromal cells in the TME

**Figure 1:**
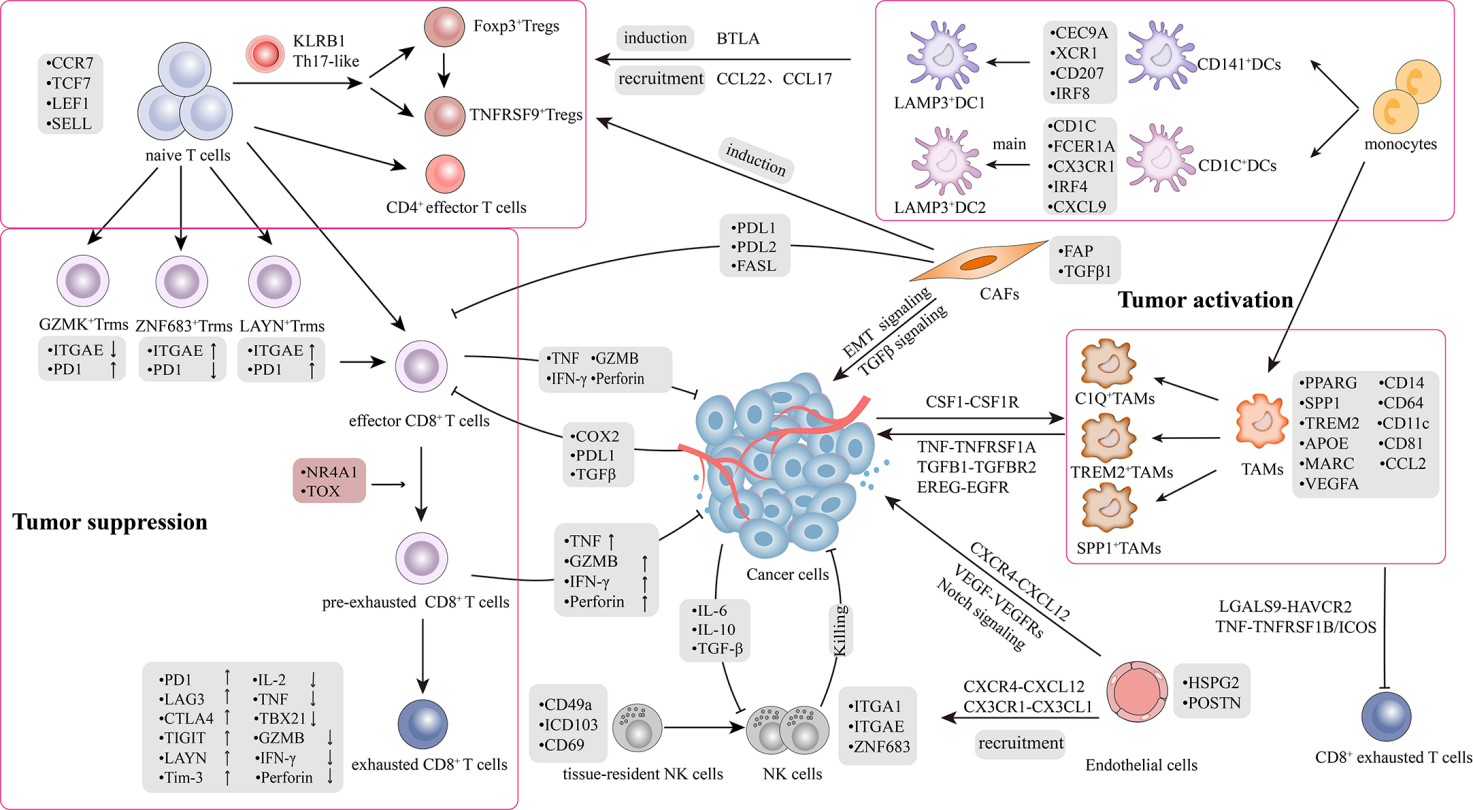
Tumor microenvironment and crosstalk in lung cancer. The picture recapitulated in-depth interaction mechanisms and signal transduction pathways between heterogeneous cell subpopulations in TME. TME comprises highly heterogeneous cancer cells and stromal cells, including T cells, B cells, natural killer cells (NKs), tumor-associated macrophages (TAMs), dendritic cells (DCs), CAFs and endothelial cells (ECs). There was a bidirectional interaction between the tumor and stromal cells within the TME. The interaction is classified into two aspects: 1) Bidirectional regulation between tumor and stromal cells; 2) Crosstalk among stromal cells.

The TME plays a significant role in tumor progression and therapeutic response in lung cancer (9, 10). The TME is defined as a complex network comprising a highly mixture of heterogeneous cancer cells and stromal cells, including a variety of immune cells (T cells, B cells, natural killer cells (NKs), tumor-associated macrophages (TAMs), and dendritic cells (DCs) and non-immune cells (CAFs) and endothelial cells (ECs)), as well as soluble growth factors, pro-angiogenic mediators, and the extracellular matrix (ECM) (11, 12). There is a bidirectional interaction between the tumor and stromal cells within the TME (13). During tumor progression, stromal cells are reprogrammed by cytokines, chemokines, and metabolites secreted by cancer cells into immunosuppressive, inactive bystanders or pro-tumorigenic phenotypes; subsequently, stromal cells assist cancer cells to promote angiogenesis and ECM remodeling, reestablishing an immunosuppressive TME. Therefore, it is essential to understand the diversity of cellular states at different stages of lung cancer and the roles that distinct subsets play during tumor progression. Despite limited experimental studies on stromal cells, scRNA-seq has enabled the characterization of TME composition and microenvironmental remodeling with unprecedented resolution (**Figure 1**).

### 2.1 Plasticity of tumor-infiltrating T cells in the TME

Tumor-infiltrating lymphocytes (TILs) play a critical role in executing anti-tumor immune responses and mediating responses to ICB treatments. The heterogeneity and plasticity of TILs have been revealed by scRNA-seq analysis, providing new strategies for cancer immunotherapy.

#### 2.1.1 Heterogeneity and dynamics of CD8^+^ T subsets

Recently, a number of scRNA-seq studies in NSCLC have clearly defined several specific subsets and delineated the progressive functional states of CD8^+^ T cells, including naive, memory, effector, effector memory T cells (Tems), tissue-resident memory T cells (Trms), and exhausted T cells (Texs) (13–19). The defining markers and potential functions of the subpopulations are listed in **Table 1**. Upon tumor neoantigen stimulation, naive CD8^+^ T cells (characterized by upregulated expression of CCR7, LEF1, TCF7, and SELL) or memory CD8^+^ T cells (characterized by high levels of ZNF683) are activated, subsequently proliferating and differentiating into effector CD8^+^ T cells (also called cytotoxic lymphocytes) to recognize and kill tumor cells (5, 14, 20). Effector CD8^+^ T cells express high levels of cytotoxic genes, including TBX21, KLF3, FCGR3A, KLRG1, and KLRB1, and exert cytotoxic activity in patients with lung cancer (14). Moreover, Tems (CX3CR1^+^CD8^+^ T cells), as a transitional state, express GZMA, GZMB, perforin 1, NKG7 (21), and low levels of inhibitory receptor genes, including PDCD1, HAVCR2, and CTLA4 (25). The subset of CX3CR1^+^CD8^+^ T cells not only exerts the ability to kill tumors, but also possesses the characteristic of memory T cells that migrate from the blood to the lungs (14, 34, 35).

**Table 1:** T Cell Subsets revealed by ScRNA-seq.

Classification	T Cell Subsets	Gene Signature	Annotation	References
CD8^+^subsets				
**Naive T cells**	naive CD8^+^ T cells	CCR7, TCF7, LEF1, SELL	Naive-like	(14)
**Effector T cells**	effector CD8^+^ T cells	TBX21, KLF3, FCGR3A, KLRG1, KLRB1/CX3CR1, PRF1, GZMA, GZMB	Teffs (cytotoxicity)	(20, 21)
**Memory T cells**	tissue-resident memory CD8^+^ T cells	CD103 (ITGAE), CD69, CXCR6, ITGA1	Tems (memory)	(14)
	CX3CR1^+^CD8^+^ T cells	High GZMA, GZMB, perforin1, NKG7 Low PDCD1, HAVCR2, CTLA4	Tems (cytotoxicity and memory)	(14)
	GZMK^+^CD8^+^ T cells	High PDCD1, low ITGAE	Trms/Texp (memory and enhanced cytotoxicity)	(14, 17)
	ZNF683^+^CD8^+^ T cells	Low PDCD1, high ITGAE	Trms/Texp (memory and enhanced cytotoxicity)	(14, 17)
	PD-1^+^TIM-3^+^ Trms	CD38, PRF1, GZMB, GZMH, PD-1, TIM-3	Trms/Texp (memory and enhanced cytotoxicity)	(17)
	LAYN^+^CD8^+^ T cells	High PDCD1, high ITGAE HAVCR2, LAG3, TIGIT	Trms/Texs (memory and impaired cytotoxicity)	(14, 17)
**Pre-dysfunctional T cells**	pre-dysfunctional CD8^+^ T cells	LAG-3, TIGIT, Low PDCD1, HAVCR2	Texp (enhanced cytotoxicity)	(22)
	CXCR5/TCF1^+^ CD8^+^T cells	TCF1	Texp (stem cell-like)	(23)
	TdLN-TTSM	TCF1, CD127, CD122, CD62L Low PDCD1	Superior anti-tumor effect	(24)
**Exhausted T cells**	Exhausted T cells	High PDCD1, CTLA4, LAG-3, LAYN, TIGIT, TIM3 Low TNF-α, IL-2, TBX21	Texs (impaired cytotoxicity)	(14, 25–29)
CD4^+^subsets				
**Naive T cells**	naive CD4^+^ T cells	CCR7, TCF7, LEF1, SELL	Naive-like	(14)
**Effector T cells**	cytotoxic CD4^+^ T cells	GZMA, GNLY, PRF1, GZMB, GZMH	Cytotoxicity	(20, 21)
**Memory T cells**	GNLY^+^CD4^+^ T cells	PRF1, GZMA, GZMB Low PDCD1, CTLA4, HAVCR2	Tems (cytotoxicity and memory)	(14, 21, 25)
	ANXA1^+^CD4^+^ T cells	ANXA1	Tcms (memory)	(14)
**Exhausted** **T cells**	CXCL13^+^CD4^+^ T cells	PDCD1, CTLA4, TIGIT, HAVCR2, BCL6, IL21, CXCL13, ICOS	Texs/TFH (impaired cytotoxicity)	(14, 25)
**Follicular Helper T Cells**	TFH	High BCL6, IL21, CXCL13, ICOS Low CXCR5	TFH (activating B cells)	(14, 25)
**T helper 17 cells**	TH17-like	KLRB1	Transitional state from naive T to Treg	(30)
**CD4^+^** **Regulatory** **T cells**	FOXP3^+^ IL2RA^+^ CD4^+^ T cells	FOXP3, IL2RA	Resting Tregs	(14, 31)
	Tregs	FOXP3, CTLA-4, PD-1, CD39, ICOS, CD38, IL32, PD-L1, PD-L2, IL2R, CCR8	Tumor-associated Tregs (immunosuppression)	(16, 20, 31, 32)
	EOMES^+^CD4^+^ T cells	EOMES	Tumor-associated Tregs (immunosuppression)	(33)
	CTLA4^+^CD4^+^ T cells	CTLA4, ICOS, TNFRSF9	Activated Tregs (immunosuppression)	(14, 31, 32)
	TNFRSF9^+^ Tregs	LAYN, REL	Activated Tregs (immunosuppression)	(14, 32)

Tcms, central memory T cells; Teffs, effector T; Tems, effector memory T cells; Trms, tissue-resident memory T cells; Texs, exhausted T; Th, T helper; TFH, follicular helper; Treg, regulatory T; Trms, tissue-resident memory T; CTLs, cytotoxic T lymphocytes; TdLN-TTSM, tumor-draining lymph node-derived tumor-specific memory T cells; CCR7, genes CC chemokine receptor 7; LEF1, lymphoid enhancer-binding factor 1; TCF7, transcription factor 7; SELL, encoding L-selectin; TBX21, T-box transcription factor; KLF3, KLF transcription factor 3; FCGR3A, Fcγ receptor IIIA; KLRG1, killer cell lectin like receptor subfamily G member 1; KLRB1, killer cell lectin like receptor B1; CX3CR1, CX3C chemokine receptor 1; PRF1, GZMA, granzyme A; GZMB, granzyme B; PRF1, perforin1; NKG7, natural killer cell granule 7; PDCD1, programmed cell death 1; HAVCR2, hepatitis A virus cellular receptor 2; CTLA4, anti-cytotoxic T lymphocyte-associated antigen 4; GZMH, granzyme H; PD-1, programmed cell death protein-1; TIM-3, T cell membrane protein 3; LAG-3, lymphocyte-activation gene-3; TIGIT, T cell immuno-receptor with immunoglobulin and ITIM domains; LAYN, Layilin; TNF-α, tumor necrosis factor alpha; IL2RA, interleukin-2 receptor subunit alpha; CCR8,genes CC chemokine receptor 8; TNFRSF9,tumor necrosis factor receptor superfamily member 9; ICOS, inducible T cell costimulatory.

ScRNA-seq has provided enriched molecular insights into the potential roles of the Trms subset in driving efficient immune responses against tumors (14, 17, 26, 36).Abundant infiltrations of Trms in the TME have been found to correlate with better clinical outcomes in lung cancer patients (17). Trms are typically defined by the tissue-resident markers CD103 (ITGAE), CD69, CXCR6, and ITGA1 and they share similar functions with CX3CR1^+^CD8^+^ Tems (5, 14). However, Trms subsets in lung cancer exhibit distinct expression patterns that can be divided into three CD8 clusters: GZMK^+^CD8^+^ (high-level PDCD1, low-level ITGAE), ZNF683^+^CD8^+^ (low-level PDCD1, high-level ITGAE), and LAYN^+^CD8^+^ (high-level PDCD1 and high-level ITGAE) (14). Notably, LAYN^+^CD8^+^ T cells express both the tissue-resident marker ITGAE and the markers of T cell exhaustion PDCD1, CTLA4, HAVCR2, LAG3, and TIGIT, suggesting a possible “exhausted” state (5). The subsets of GZMK^+^CD8^+^ and ZNF683^+^CD8^+^ with lower exhaustion scores than LAYN^+^CD8^+^ may represent clusters of “pre-exhausted” T cells based on monocle trajectory analysis (14). Furthermore, another study has revealed a distinct subset, PD-1^+^TIM-3^+^ Trms, characterized by the expression of markers associated with cytotoxic function (PRF1, GZMB, and GZMH), T cell activation (CD38), and high levels of PD-1 and TIM-3 (17). The PD-1^+^TIM-3^+^ Trms subset was enriched in responders to PD-1 inhibitors, suggesting that PD-1 is not only a symbol of functional exhaustion (17). In conclusion, the differential functional status of Trms subsets indicates that Trms should not be considered as a homogeneous population. Instead, they represent a functional pattern that is different from conventional Trms within tumors. This overlap creates difficulties in the specific definition and functional classification of cell subsets; therefore, reliance on a single marker to define a cell subset is insufficient and further studies are required to establish precise definitions of these subsets.

T cell exhaustion, characterized by the loss of effector and memory functions, has been extensively studied for its role in immunodeficiency (37). The subset of exhausted (or ‘dysfunctional’) CD8^+^ T cells (Tex) is characterized by high expression levels of inhibitory receptor genes such as PDCD1, CTLA4, LAG-3, LAYN, TIGIT, and TIM3, and low expression levels of tumor necrosis factor alpha (TNF-α), interleukin-2 (IL-2), and T-box transcription factor (TBX21) (5, 14, 26–29). It is well-known that exhausted T cells gradually lose their cytotoxic ability, which is considered a key mechanism of tumor immune evasion and primary factor for non-response to immunotherapy (22, 25). However, the mechanisms that induce the formation of abundant exhausted T cells are complex and remain unclear, which require further investigation. Collectively, the heterogeneous subsets of CD8^+^ T cells may correspond to the processes of continuous response to the TME, exhibiting diverse functional states.

#### 2.1.2 Gradual dysfunctional process of CD8^+^ T cells

The inferred developmental trajectory based on transcriptional relatedness suggested a branched structure in which the LAYN^+^CD8^+^ exhausted, LEF1^+^CD8^+^ naive, and CX3CR1^+^CD8^+^ effector subsets are located at the ends of different branches, with GZMK^+^CD8^+^ T cells and ZNF683^+^CD8^+^ T cells positioned in between them (14). This path supports the previously discussed observation of the existence of CD8^+^ T cells in an intermediate functional state and suggests that CD8^+^ T cells undergo gradual differentiation (5, 26).

Tex cells are considered as dysfunctional T cells with impaired cytotoxicity and are strongly associated with clinical non-response to ICB therapy in lung cancer patients (5, 14, 21–23, 25, 37). The presence of low levels of dysfunctional T cells may be critical for ICB to provide durable benefit (20). Recent research has identified a four-stage developmental model from precursor exhausted T (Texp) cells to Tex cells, accompanied by a gradual decrease in T cell factor family member (TCF1) (21, 25).Notably, the subset of PD-1^+^TCF-1^+^CD8^+^ Texp has been considered as stem cell-like CD8^+^ T cells that respond well to ICB therapy in responsive tumors (21, 25). Similarly, transcriptional profiling has shown that Texp cells may overlap with CXCR5/TCF1^+^CD8^+^T cells, which correlates with a durable response to ICB in multiple tumors (25, 34, 36). Moreover, a mouse study revealed similar pre-dysfunctional CD8^+^ cell patterns characterized by LAG-3 and TIGIT, but with low levels of PDCD1 and HAVCR2 (22). Interestingly, in another mouse tumor model, a population defined as tumor-draining lymph node-derived tumor-specific memory T cells (TdLN-TTSM) was identified. The TdLN-TTSM cells express high levels of TCF, memory molecules (CD127, CD122, and CD62L), and low levels of PD-1 (24). This subset was confirmed to be upstream of Texp cells differentiation and further differentiated into TCF1^+^TOX^+^ Texp and TCF1^-^TOX^+^ Tex cells, which were able to effectively migrate into the TME (24). Compared with Texp cells, TdLN-TTSM cells exhibited stronger proliferation potential as well as stronger anti-tumor ability, making them a promising target for cancer immunotherapy.

In conclusion, phenotype switching leads to functional changes in CD8^+^ T cells. In the early stages of lung cancer, effector CD8^+^ T cells are the most abundant, whereas the proportion of exhausted CD8^+^ T cells is much higher in advanced lung cancer (14). The effector/exhausted ratio in the early stages of lung cancer is intermediate between that of normal lung tissue and advanced stages, revealing the dynamic evolution of CD8^+^ T cell subsets during lung cancer progression (19). These findings demonstrate that T cell dysfunction is a continuous process rather than an isolated state, and the dysfunctional CD8^+^ T cells in the TME should not be considered nonfunctional, but T cells with novel functions. Consequently, further researches on Texp and TdLN-TTSM using scRNA-seq are important for optimizing T cell-based lung cancer therapy and combined therapy with ICB.

#### 2.1.3 Heterogeneity and dynamics of CD4^+^ T cells

The application of scRNA-seq provides an in-depth view of the CD4^+^ T cells map. The developmental trajectory has shown that differentiation pathways generate distinct CD4^+^ subsets, including naive, Tems, central memory T (Tcms), and Tex CD4^+^ T cells. Naive CD4^+^ T cells are characterized by high expression of CCR7, TCF7, LEF1, and SELL; Tems CD4^+^ T cells are characterized by the GNLY^+^CD4^+^ T cluster expressing PRF1, GZMA, and GZMB and lower signatures of PDCD1, CTLA4, and HAVCR2; Tcms CD4^+^ T cells are defined as ANXA1^+^CD4^+^ T cells (14). In addition, the CXCL13^+^CD4^+^ T cell subset indicated in lung cancer has been described by high expression of exhaustion markers, including PDCD1, CTLA4, TIGIT, and HAVCR2, which is considered as an exhausted CD4^+^ T cell subpopulation (14). T follicular helper cells (TFH) may be an important constituent of tertiary lymphoid structures (TLSs) at the tumor site, contributing to the intratumoral immune response of CD8^+^ T and B cells (30, 33).

Conventional CD4^+^ T cells, which promote anti-tumor immune responses and cytotoxic effects, inhibit tumor growth by secreting interferon-γ (IFN-γ) and tumor necrosis factor (TNF), whereas tumor-resident Tregs have been shown to suppress the infiltration and anti-tumor activities of effector CD8^+^ T cells (26). For non-Treg CD4^+^ T cells, the developmental trajectory shows that naive CCR7^+^CD4^+^cells are located at the root, the effector GNLY^+^CD4^+^ and exhausted CXCL13^+^CD4^+^ cells are at the developmental end, and the intermediate subgroups are mostly transitional states such as ANXA1^+^CD4^+^, CD69^+^CD4^+^, EOMES^+^CD4^+^, and GZMA^+^CD4^+^ (14). However, these subtypes are not present in all tumor types. Interestingly, the CXCL13^+^CD4^+^ T cell population expresses similar characteristics, such as high expression of BCL6, IL21, CXCL13, and ICOS, and low expression of CXCR5 with TFH, indicating that exhausted CD4^+^ T cells may convert to the TFH phenotype (14). Treg and TFH cells show high levels of proliferation in tumors, reflecting their potential roles in the intratumoral CD4^+^ T cell response (5). Developmental trajectories of scRNA-seq have revealed that T helper 17 (Th17) cells are located in the midst of differentiation from naive CD4^+^ T cells to Tregs, representing a transitional state (30).With the development of lung cancer, the proportion of effector CD4^+^ T cells gradually decreases, whereas the proportion of exhausted CD4^+^ T cells considerably increases. This change indicates the gradual exhaustion of effector CD4^+^ T cells (19).

#### 2.1.4 Heterogeneity of regulatory T cells

Tregs, which contribute to immunosuppression and tumor progression, are a vital subpopulation of CD4^+^ T cells. Recent studies have shown that Tregs undergo phenotypic switching to expand under the stimulation of tumor cells, rather than by the proliferation of existing Tregs (5). Therefore, scRNA-seq technology has been employed to study CD4^+^ Tregs, which can provide insights into the TME. Tumor-associated Tregs highly express immunosuppressive molecules, including FOXP3, CTLA-4, PD-1, CD39, ICOS, CD38, and IL32, as well as several specific surface signaling molecules (PD-L1, PD-L2, interleukin-1 receptor 2, and chemokine CCR8) (16, 20). Although FOXP3 is regarded as a classic hallmark of Tregs, scRNA-seq has revealed a distinct subset of FOXP3^-^CD4^+^ T cells, that are immunosuppressive in the TME and share similar functions with FOXP3^+^CD4^+^ Tregs (19, 38, 39). Additionally, an intriguing study also identified EOMES^+^CD4^+^ T cells with immunosuppressive effects similar to FOXP3^+^ Treg cells (33). The elaborate landscape depiction of Tregs provides insight into the synergistic effect of targeted and ICB therapy.

The description of Tregs differentiation status is variable. Studies have identified two distinct groups of Tregs: FOXP3^+^IL2RA^+^CD4^+^ and CTLA4^+^CD4^+^ Tregs (31). The former has been described as resting Tregs; whereas the latter are known as activated Tregs (suppressive Tregs). FOXP3^+^CD4^+^ Tregs mostly exist in the blood, whereas CTLA4^+^CD4^+^ Tregs clone and amplify locally within tumors, indicating local clonal expansion of tumor-resident Tregs. The suppressive CTLA4^+^CD4^+^ Treg cell subsets are also heterogeneous. Compared to the Treg cells in blood or adjacent normal tissue, Tregs in the tumor express higher levels of CTLA4, inducible T cell costimulator (ICOS) and tumor necrosis factor receptor superfamily member 9 (TNFRSF9; encoding 4-1BB), which reflect an activated state (32). Thus, the expression level of TNFRSF9 also reflects the existence of two distinct groups of Tregs: resting and activated. TNFRSF9^+^ Tregs highly express genes related to immunosuppressive functions, such as IL1R2 (14), LAYN and REL (26), suggesting that they are a major component of functional tumor Tregs. The transformation of CD4^+^ T cells into Tregs is a continuous process associated with tumor development. The developmental trajectory shows a gradual transition from TNFRSF9–Treg (resting) to TNFRSF9^+^ Treg (activated) cells. Moreover, scRNA-seq analysis has revealed a transitional phenotype, Th17-like CD4^+^ T cells, characterized by the high expression of KLRB1, which is a transitional state in the conversion of naive CD4^+^ T cells to Tregs (26).

In conclusion, CD8^+^ and CD4^+^ T cells exhibit significant heterogeneity and perform various functions in the TME. Tumor cells and their secretory mediators reprogram T cells to differentiate into immunosuppressive phenotypes, including Texs and Tregs, thereby contributing to tumor progression. Their dynamic transition from tumor-killing to tumor-promoting states poses a major challenge for understanding the interactions between different subpopulations and developing effective immunotherapeutic strategies.

### 2.2 Heterogeneity of tumor-infiltrating B cells

B cells are an essential component of the adaptive immune system and are responsible for promoting anti-tumor immune responses (40). In NSCLC, tumor-infiltrating B cells have been categorized into multiple heterogeneous subpopulations with distinct functional and surface markers (15, 19, 27, 41). They are represented as mucosa-associated lymphoid tissue-derived B (MLAT), germinal center (GC) B, follicular B, and plasma B cells. The most studied subset of follicular B cells was divided into naive follicular B (CD20^+^CD27^−^IGHD^+^) and memory follicular B cells (CD20^+^CD27^+^IGHD−). Similar to the subset of naive follicular B cells, another cohort identified naive-like B cell clusters in NSCLC with high levels of CD19, CD22, MS4A1 (CD20), TCL1A, and CD83 (41), which were mainly located in the TLSs of lung tumor tissues and significantly decreased with tumor progression. High infiltration levels of naive-like B cells were associated with better relapse-free survival and overall survival. Consequently, the presence of B cells in TLSs is beneficial for protective immunity in lung cancer patients (42–45). Furthermore, plasma B cells express marker genes CD38, TNFRSF17(BCMA), and IGHG1/IGHG4, which inhibit cell growth in the early stage of NSCLC but play the opposite role of promoting cell growth in advanced tumors, thus exhibiting heterogeneity in lung cancer progression. Recent studies have shown that intratumoral plasma cells can predict the outcome of PD-L1 blockade in NSCLC (46). Overall, scRNA-seq studies have revealed the phenotypic and functional heterogeneity of B cell subsets and their potential roles and mechanisms in lung cancer progression from a general perspective. However, the function of B cell subsets in TLSs and their role in immunotherapy remain to be elucidated.

### 2.3 New advances in defining TAM subsets

TAMs, a dominant myeloid subpopulation in the TME, have traditionally been divided into M1-like (pro-inflammatory) or M2-like (anti-inflammatory) subsets based on their distinct activation factors and effector functions (47). However, dichotomous classifications are now oversimplified and outdated due to the broad spectrum of macrophage plasticity revealed by scRNA-seq studies. Recently, specific cellular molecules in unique subsets of macrophages have received considerable attention.

Accumulating scRNA-seq studies have identified several representative subsets of TAMs in NSCLC, defined by the symbolic markers complement 1 q (C1Q), SPP1, triggering receptor expressed on myeloid cells-2 (TREM2), NLRP3, LYVE1 (48–52). Protumorigenic macrophages consist of C1Q^+^, SPP1^+^, and TREM2^+^ subsets (48, 49, 52, 53). C1Q^+^ TAMs are thought to be immunosuppressive in the TME and are associated with T cell exhaustion and a poor prognosis for lung cancer (54–57). In addition, SPP1^+^ TAMs exhibit high expression of genes associated with angiogenesis, such as VEGFA, CXCL8, VCAN, and ANGPTL4, and loss of MHC class II expression. SPP1^+^ and C1Q^+^ TAMs exhibit higher levels of M2-like signals and remodel the TME in different ways. SPP1^+^ TAMs have a stronger interaction with CAFs and ECs, thereby remodeling the TME. In addition, studies have demonstrated that high SPP1 expression in macrophages promotes lung cancer invasiveness and is associated with a worse clinical outcome in multiple cancer types (48, 51). TREM2^+^ macrophages are stably enriched in both early and late stages of lung cancer and have been defined as a pro-tumorigenic subset (48, 49). Additionally, NLRP3^+^ and LYVE1^+^ macrophages may exert anti-tumor effects in the TME. NLRP3^+^ and LYVE1^+^ macrophages likely represent two clusters of pro-inflammatory tissue-resident macrophages (TRMs) enriched in non-cancerous tissues. Unlike NLRP3^+^ macrophages, LYVE1^+^ macrophages have the potential to develop into C1Q^+^ TAM and reprogram the TME (48). Although scRNA-seq studies have provided insights into the potential functions of macrophage subsets, formal evidence is limited and requires further investigation.

Notably, TRMs subsets are also pivotal cellular components that mediate the innate immune response and permanently reside in the lung tissue, playing crucial roles in tissue homeostasis and inflammation control (17, 58). Studies have demonstrated that TRMs may contribute to tissue remodeling and tumor cell invasion during the early tumor stages. In advanced tumor, TRMs are redistributed and significantly reduced during tumor growth. Moreover, the population of CD4^+^ and effector CD8^+^ T cells increases and the expression of PD-1 decreases in TRM-exhausted lesions, which in turn impairs the survival and growth of early tumor cells (59). Overall, TRMs play a tumor-promoting role in early stages of NSCLC.

Immune remodeling against macrophages is initiated in the early stages of tumors and perpetuates during tumor progression (15, 16, 19, 60). In early stages of lung cancer, TAMs exhibit significant immunosuppressive activity compared to normal tissue-derived macrophages. They express high levels of the immunomodulatory transcription factors PPARg, CD14, CD64, and CD11c and low levels of CD86 and CD206 (32). Moreover, higher levels of IL-6 have been detected in intrapulmonary macrophages, which significantly contributes to tumor aggressiveness (61). These findings suggest that macrophages play a suppressive role in early stages of lung cancer. Additionally, TAMs express higher levels of CD81, TREM2, apolipoprotein E (APOE), and macrophage receptor with collagenous structure (MARCO), which have been associated with poor survival rates in patients based on TCGA database (32, 62). Several early scRNA-seq studies reported that APOE, and SPP1 in TAMs promote tumor growth and metastasis (16, 60, 63). In the advanced stages of lung cancer, the proportion of subsets with high expression of immunosuppressive markers is upregulated and persists (19, 30). For example, a group of VEGFA^+^ TAMs highly expressed hypoxia-inducible genes such as SLCA2, HK2, ANGPTL4, and VEGFA, which is beneficial for tumor angiogenesis (19). The steady enrichment of TREM2^+^ TAM subsets in tumors indicates that TAMs can inhibit anti-tumor immune responses, providing potential targets for immunotherapy (20). Collectively, these studies suggest that the application of scRNA-seq may help to develop a more comprehensive profile of TAMs in lung cancer and provide more precise strategies for immunotherapy.

### 2.4 The functional reshaping of NK cells

Under the influence of TME, the tumor-killing function of NK cells is gradually impaired and develops into an exhausted state with the upregulation of surface inhibitory markers (19, 30, 32). scRNA-seq studies have revealed the transformation of NK cell subsets in different stages of tumors and identified new clusters (19, 30, 31). NK cells are commonly divided into CD16^+^ and CD16^−^ populations, with the CD16^+^ NK cluster showing high expression of transcripts encoding fibroblast growth factor binding protein 2 (FGFBP2) and CX3CR1, both of which are involved in promoting lymphocyte cytotoxic functions. In contrast, the CD16^−^ NK cluster upregulates tissue-resident markers such as ITGA1, ITGAE, and ZNF683 (30). The proportion of NK cells with different functions gradually changes during the occurrence and development of lung cancer. Compared with the early stage of lung cancer, CD16^+^ NK cells were decreased, and the CD16^−^ NK cluster was highly enriched in advanced NSCLC (30). In addition, a population of tissue-resident NK (trNK) referred to as intraepithelial ILC1 (ieILC1), has been defined as CD56^+^CD16^-^CD127^-^tBET^+^EOMES^+^ cells and can be recognized by the expression of CXCR6 and resident markers, including CD49a, CD103, and CD69 (64, 65). IeILC1 exerts potent anti-tumor effects by producing granzyme B and perforin. In addition, stimulation with IL-15 or IL-18 can increase their ability to produce IFN-γ (66). Thus, iEILC1-like cell-infiltrating tumors may be associated with greater anti-tumor function (67).

Although NK cell subsets with cytotoxic effects exist in the TME, the NK cell toxicity is impaired during tumor progression. The immunosuppressive effects of the TME directly impaired the viability of NK cells (68). However, various factors in the TME, including IL-6, IL10, and transforming growth factor-β (TGF-β), indirectly led to the inhibition of NK cell viability by disrupting the balance between the activation and inhibition signals (69). Exhausted NK cells are often observed to upregulate inhibitory receptors such as PD-1 (70) and TIM-3 (71) and decrease cytolysis and cytokine secretion capacity (72). Moreover, a study on the co-expression of PD-1 and TIM-3 in NK cells is reported in colorectal, melanoma, and bladder cancers (73). It was demonstrated that increased Tim-3 expression in NK cells was associated with NK cells exhaustion and predicted a poorer prognosis, while Tim-3 blockade ameliorated NK cell-mediated cytotoxicity in human lung cancer (71). However, there is a need for further research to explore the connection between PD-1 and TIM-3 and their role in promoting NK exhaustion in lung cancer. To some extent, the phenotype of exhausted NK cells overlaps with that of trNK cells, indicating that trNK may be further transformed into the exhaustion phenotype (65). Although the heterogeneity and functions of NK cells have been partially revealed, the dynamics of NK cell dysfunction in the TME still require further investigation. In summary, expansion or reactivation of NK cells may serve as a therapeutic strategy to restore anti-tumor immunity in lung cancer.

### 2.5 Phenotype reprogramming of DCs

Dendritic cells are crucial for the induction and maintenance of anti-tumor immunity as professional antigen-presenting cells (74, 75). ScRNA-seq studies of lung cancer have revealed the phenotypic and functional heterogeneity of DCs, which may include both immunostimulatory and immunosuppressive subsets. Several scRNA-seq studies have been conducted to describe the tumor-infiltrating DC landscape to reveal its remodeled function in cancer. DCs can be clustered into conventional DCs (cDCs), plasmacytoid DCs (pDCs), and activated DCs, with cDCs further divided into CD141^+^ DCs and CD1C^+^ DCs (15, 32, 76, 77). In scRNA-seq studies of lung cancer, CD141^+^ DCs clusters were revealed with marker genes of CLEC9A, XCR1, CD207, IRF8, and CD1C^+^ DCs clusters highly expressed CD1C, CX3CR1, FCER1A, and IRF4. The proportion of CD141^+^ DCs is significantly lower in the tumor site than in the normal lung and is associated with the formation of TLSs (16, 32). Conversely, CD1C^+^ DCs were enriched in tumor tissues and expressed higher levels of CCL17 and CCL22, which can recruit Tregs to induce immunosuppression in the TME (32). Notably, the LAMP3^+^ DCs cluster was identified as an activated and mature DC subset with high expression of the maturation markers CCR7, CD83, CD40, and RELB, along with the immunoregulatory molecules CD200, CD274, FAS, and ALDH1A2, as well as low expression of Toll-like receptor (TLR) signaling genes (15). LAMP3^+^DCs from different sources have heterogeneous phenotypes and functions. Both CD141^+^ DCs and CD1C^+^ DCs in tumors have the potential to differentiate into LAMP3^+^ DCs upon uptake of tumor antigens, although more CD141^+^ DC-derived LAMP3^+^ DCs have been observed in lung cancer (50, 78). CD141^+^ DC-derived LAMP3^+^ DCs highly expressed IL12B, a specific molecule that induces the differentiation of Th1 cells (79). It also expressed B and T lymphocyte attenuator (BTLA), which can induce the differentiation of Tregs and affect Treg cell-mediated immune tolerance (80), in accordance with the ability of mature DCs expressing high levels of immunoregulatory molecules (mregDCs) to facilitate the differentiation of naive CD4^+^ T cells into Tregs (78). In contrast, CD1C^+^ DC-derived LAMP3^+^ DCs expressed high levels of marker genes CD1E and CCL17, which can recruit CCR4^+^ Tregs into tumors and contribute to immunosuppression (81). Interestingly, the CXCL9^+^CD1C^+^ DCs cluster predominated over other clusters in the tumor tissue and was more likely to differentiate into LAMP3^+^ DCs, thus enhancing the immunosuppressive effect. This process was accompanied by a decreased expression of CXCL9 and increased levels of IDO1. Notably, in another study, the LAMP3^+^ DCs cluster was similar to a cluster of mregDCs and shared similar gene expression (48). Collectively, LAMP3^+^ DCs from different sources retained the corresponding features of their specific transcriptomic properties, which diversified their functions, while sharing analogous maturation signatures. Another distinct pDCs cluster was identified to promote adaptive immunity in the TME (16, 32, 50, 82). It is marked by upregulation of leukocyte immunoglobulin-like receptor (LILR) family genes, GZMB, and low expression of CD86, CD83, CD80, and LAMP3 activation marker (82–84).

Overall, DCs mainly exert immune-promoting functions. However, tumor-infiltrating DCs undergo marked and prolonged remodeling during tumor development, reshaping the TME and promoting tumor growth. Further studies are required to fully elucidate their role in cancer immunity and immunotherapy, especially in terms of the complex co-expression pattern of activating and inhibitory molecules.

### 2.6 Extensive tumor-promoting characteristics of CAFs

CAFs are unique stromal cells that play an important role in tumor proliferation, invasion, metastasis, and anti-tumor therapies (84). Fibroblasts are a heterogeneous population whose phenotypes are easily influenced by the mediators in the TME (85, 86). The immune remodeling effect of the TME causes CAFs to be immunosuppressive through high expression of some specific molecules, interactions between receptor and ligand, and activation of signaling pathways. Studies have found that in early stage lung cancer nodules, the CFD^+^CXCL14^+^ CAFs (31) and ADH1B^+^ CAFs (87) were the most abundant. The CFD^+^CXCL14^+^ CAFs expressed CXCL14 and CXCL12 which are enriched in signaling IFN-γ responses and IL2-STAT5 signaling pathways, indicating their potential immunomodulatory role. The ADH1B^+^ CAFs were found to show high expression of the retention genes CCL19, CCL21 and vascular cell adhesion molecule 1 (VCAM1), which are believed to play a role in T cells recruitment. Moreover, these cells were located in the TLSs, suggesting they may have an antigen presentation role. Promoting the differentiation of CAFs into the ADH1B^+^ subtype may improve the anti-tumor immune effect (87). With the tumor progression, the FAP^+^TGF-β^+^ CAFs cluster and FAP^+^α-SMA^+^ CAFs were specifically enriched in advanced tumors, with high expression of the characteristic factors FAP, TGF-β, PDPN, activation related genes COL1A1, COL3A1 and BGN and some unique tumor-associated collagens such as collagens V, VIII, and XII, which are associated with tumor growth (16, 19, 27, 30). Furthermore, hallmark pathway analysis confirmed that the angiogenic-oxidative phosphorylation, epithelial-mesenchymal transition (EMT), and TGF-β signaling supporting tumor progression were highly activated in CAFs. CAFs can be transformed from myofibroblasts in tumor tissues and display high activity of PDGF, JAK/STAT signaling, TGF-β, and hypoxia-induced pathways, which contribute to extracellular matrix, remodeling, angiogenesis, and tumor progression. Interestingly, CFD^+^CXCL14^+^ fibroblasts expressed MCH class II molecules and CD74 related to antigen-presenting function on the surface, which may be considered as a new type of CAFs (19, 88). In conclusion, several studies focusing on CAFs have demonstrated that CAFs may contribute to tumor progression by regulating angiogenesis, extracellular matrix synthesis, and remodeling, suggesting that CAFs may be feasible therapeutic targets for anti-tumor immunotherapy.

### 2.7 Angiogenesis and matrix remodeling properties of ECs

As important stromal cells in the TME, ECs are associated with angiogenic functions and play an essential role in tumor initiation and progression (89). The nomenclature of ECs varies in different studies and can be divided into tumor ECs and non-malignant ECs. In this review, we focused on the heterogeneity of tumor ECs. Regarding tumor ECs, one study identified two populations, IGFBP3^+^ ECs and SPRY1^+^ ECs (27), another study defined IGFBP7^+^PLVAP^+^ as tumor ECs, with high expression of HSPG2 and POSTN, which are associated with angiogenesis (19), and another study suggested that tumor ECs highly express insulin receptor (INSR) and HSPG2. Similarly, another study also identified two types of tumor ECs: INSR^+^ tumor EC (INSR^hi^HSPG2^+^PLVAP^+^) and ACKR1^+^ tumor EC (ACKR1^+^SELP^+^IL1R1^+^) in early-stage lung cancer that radiologically manifests as part-solid nodules (31). Despite the differences in markers across studies, they demonstrate similarities in pathway analysis and function in diverse subsets. Hallmark pathway analyses confirmed that the CXCL12-CXCR4 pathway, VEGF, Notch signaling, and metabolic pathways related to angiogenesis and extracellular remodeling were highly activated in the tumor ECs (30, 90). In addition, EC subsets show significant heterogeneity at different stages of lung cancer development, as revealed by scRNA-seq (16, 19). In early-stage lung cancer nodules and metastatic lung cancer, IGFBP7^+^PLVAP^+^ tumor ECs were greatly amplified, whereas the proportion of extra-alveolar capillary ECs (cECs), EDN1^+^CCL2^+^ ECs, decreased. It is known that EDN1^+^CCL2^+^ ECs overexpress genes related to lymphocyte activation and homing ability, such as BIRC3, CCL2, CD44, and ICAM1. Collectively, tumor ECs are remodeled to decrease their activities of lymphocyte cell activation and homing and increase their ability of angiogenesis and matrix remodeling, contributing to tumor immune tolerance.

## 3 Intercellular network and functional crosstalk in the TME

The advent of scRNA-seq technology has provided new insights into the complex cellular interactions and signaling pathways within the TME, thereby aiding in the identification of potential immunotherapy targets. Several tools, such as “cellphone DB” and iTALK have been established to infer putative cell-to-cell interactions based on ligand-receptor signaling from high-resolution scRNA-seq data (20, 91–97). In this review, we summarize the key findings regarding cell subpopulation interactions from the existing studies.

### 3.1 Bidirectional regulation between tumor and stromal cells

Increasing evidence suggests that cancer cells modify their immunogenic properties and remodel adjacent stromal cells to create an immunosuppressive microenvironment that favors their survival and proliferation (5, 14, 15, 27, 48, 98).

Recent scRNA-seq studies have revealed that tumor cells regulate the expression of relevant carcinogenic molecules which contribute to immune escape (99, 100). CD47, a classic immune escape molecule, prevents immune cell maturation and the release of immune activation factors through the CD47-SIRPA axis. A scRNA-seq study further confirmed that CD47 and SIRPA were upregulated in lung cancer compared to normal lung tissue (51). Additionally, it was found that CD24 was found to be generally elevated in the epithelial cells of lung cancer. High levels of CD24 were associated with shortened overall survival (OS) and progression-free interval (PFI), suggesting that CD24 may be a viable target for treatment of lung cancer (20). Furthermore, the ligand-receptor pairs of GRN and TNFRSF1A were observed to play a significant role in the communication between cancer stem cells and myofibroblasts. VEGFC, an activator of lymphatic vessel formation, has been considered as a risk factor for lung cancer and has been associated with a poor prognosis in lung cancer patients (97). In addition, the gene AKR1B1 has been reported to be upregulated in mixed-lineage lung cancer cells. AKR1B1 plays an important role in tumorigenesis and may serve as a candidate target for the treatment of lung cancer patients (98). SFTPC, which encodes pulmonary surfactant-associated protein C, is responsible for maintaining the stability of lung tissue. Studies have determined that SFTPC is overexpressed in long-term surviving NSCLC patients, indicating its potential as a biomarker for lung cancer treatment (101).Furthermore, activation of the BTLA/HVEM pathway is regarded as the initiator of immune escape in lung cancer (102, 103).

Tumor cells orchestrate stromal cells to favor immunosuppressive environment reconstitution, including TAMs, CAFs, and ECs (30, 31, 99, 104, 105). Previous studies have shown that tumor cells achieve immune evasion through a variety of mechanisms, including the downregulation of tumor antigens, low expression of MHC molecules, a lack of costimulatory molecules, and activation of the Fas/FasL pathway (84, 99). ScRNA-seq studies have enriched the mechanisms of immune evasion; for instance, activation of signaling pathways between tumor cells and suppressive cell subsets leads to phenotypic remodeling and promotes the malignant proliferation of lung cancer. The interaction between tumor cells and monocyte-macrophages (mo-Macs) is significant, especially for the activation of the growth factor signaling pathways VEGFA and VEGFB. The mo-Macs were predicted to deliver activation signals through the TNFR (TNF-TNFRSF1A), TGFBR (TGFB1-TGFBR2), and EGFR (EREG-EGFR) pathways to tumor cells. In turn, the mo-Macs received their own growth signals through the CSF1-CSF1R pathway (15). Furthermore, the Sema3A/Neuropilin-1 signaling axis has been confirmed to recruit TAMs to hypoxic sites (105, 106); thus, targeted impairment of this pathway can hinder the migration of TAMs to the TME. Additionally, tumor cells interact with tumor ECs through angiogenic signals, including VEGF-VEGFRs, Eph/ephrin, and the CXCL12-CXCR4 pathway. These signals reshape ECs towards immunosuppression and contribute to extracellular matrix remodeling, angiogenesis, and tumor progression (13, 26, 101). Hallmark pathway analysis has revealed that CAFs display significant activation of several key signaling pathways, including angiogenesis, oxidative phosphorylation, EMT, platelet-derived growth factor (PDGF), fibroblast growth factor (FGF) growth signaling pathways, as well as TGF-β signaling. These pathways contribute to the extensive tissue remodeling, angiogenesis, and tumor progression (30). The intricate interaction between tumor cells and stromal cells in the TME provides insights into targeted therapeutic strategies, such as blocking surface markers or specific signaling pathways to reactivate certain subpopulations and support immune checkpoint responses.

### 3.2 Crosstalk among stromal cells

The phenotypic remodeling of immunosuppressive cells promotes tumor progression, and is also directly connected to the T cell immune response. The mo-Macs have been shown to deliver inhibitory signals (LGALS9-HAVCR2 and TNF-TNFRSF1B/ICOS) to exhausted CD8^+^ T-cells (15). Interestingly, a subset of M1hot TAMs (M2-like TAMs co-expressed with a strong/hot M1-like signature) recruits Trms by expressing CXCL9 and providing energy to Trms through possibly a mechanism of fatty acid uptake. The ratio of M1hot TAMs is associated with better outcomes, thus providing novel therapeutic approaches to recover adaptive anti-tumor responses by reprogramming TAMs to the M1hot phenotype. Moreover, the ligand-receptor pair consisting of EGFR and AREG was the key ligand-receptor pair in the communication of M1 macrophage with other types of cells. In addition, the mature subsets of LAMP3^+^ DCs and mregDCs interact with T lymphocyte cells by overexpressing BTLA to promote the differentiation of naive CD4^+^ T cells into Tregs, thus sponsoring immune tolerance (48). Another study also showed that the interaction between Treg and DCs is a central mechanism for maintaining the immune tolerance microenvironment. Compared to naive T cells, Treg cells have stronger and longer interactions with DCs (94). Furthermore, a cluster of CAFs was discovered using scRNA-seq to drive resistance to immunotherapy by upregulating the protein levels of PD-L1 and CTLA-4 in Treg cells (107). In addition, ECs strongly express the ligands CXCL12 and CX3CL1, which can interact with the receptors of CXCR4 and CX3CR1 on NK cells, respectively, revealing that ECs may contribute to the recruitment of NK cells (16, 19). Lymphocyte-EC interactions with respect to lymphocyte recruitment and homing pathways, such as ICAM1/2-integrin and HAS2/MMP7-CD44, were enriched in early stages of lung cancer nodules compared to advanced lung cancer, indicating that EC remodels lymphocytes in the direction of immunosuppression (19). The interaction among stromal cells is highly complex but can be uncovered using scRNA-seq, proposing more possible therapeutic strategies for immunotherapy.

## 4 Recent advances in spatial transcriptomics utilization in lung cancer

Although scRNA-seq has been a valuable tool in the study of lung cancer, it has its limitations in providing spatial information, which is critical for a comprehensively understanding the progression and intercellular interactions. To overcome these limitations, spatial transcriptomics (ST) has emerged as a cutting-edge technology that seamlessly integrates with scRNA-seq, enabling the study of gene expression patterns within their spatial context. ST can map gene expression to specific locations within tissue, enabling the study of gene expression patterns within their spatial context. In the context of lung cancer, this technique has demonstrated great potential for gaining a deeper understanding of the heterogeneity of the tumor microenvironment, revealing the interactions between cell subpopulations behind spatial organization patterns (52, 108–111). Recent developments in the application of spatial transcriptomics in lung cancer research are discussed below.

ST provides a deeper understanding of the interactions between cell subpopulations behind spatial organization patterns. Tertiary lymphoid structures (TLSs), a heterotopic structure that aggregates T and B lymphocytes, have been associated with increased local immune reactions; however, traditional research methods have limited our understanding of this structure. Recent studies utilizing ST have further investigated the structure of TLSs and identified that CXCR5 and CD79 are markers associated with TLSs. CXCR5 expression on B cells is a characteristic of subpopulations migrating to TLSs. TLSs are often located outside tumor nests, with higher densities of B cells and CD4^+^ T cells than inside or around tumors (108). Additionally, studies have shown that CD4^+^ T cells, CD20^+^ B cells, and CD38^+^ T cells are found in close proximity, contributing to the formation of TLSs-like structures (109). Interestingly, studies have shown that in addition to TLSs, a large number of immune-rich areas (immune hotspots) without germinal center-like structures are observed in the tumor and peritumor areas of NSCLC. In these immune hotspots, the interaction between CD20^+^CXCR5^+^ B cells and Treg cells is enhanced. In contrast, compared to immune-rich areas in the peritumor region, the density of CD20^+^CXCR5^+^ and CD79b^+^ B cells in immune-rich areas in the tumor center is significantly lower, and the diversity of immune cell interactions is lower (108).

Therefore, the integration of single-cell transcriptomics with ST strategies holds great potential to improve the accuracy of the TME characterization and to advance the development of novel diagnostic and therapeutic approaches. However, despite its promising applications, the current knowledge on spatial transcriptomics in lung cancer is limited. Thus, further experimental researches are required to complement the existing evidence and to elucidate the full potential of this innovative approach.

**Figure 2:**
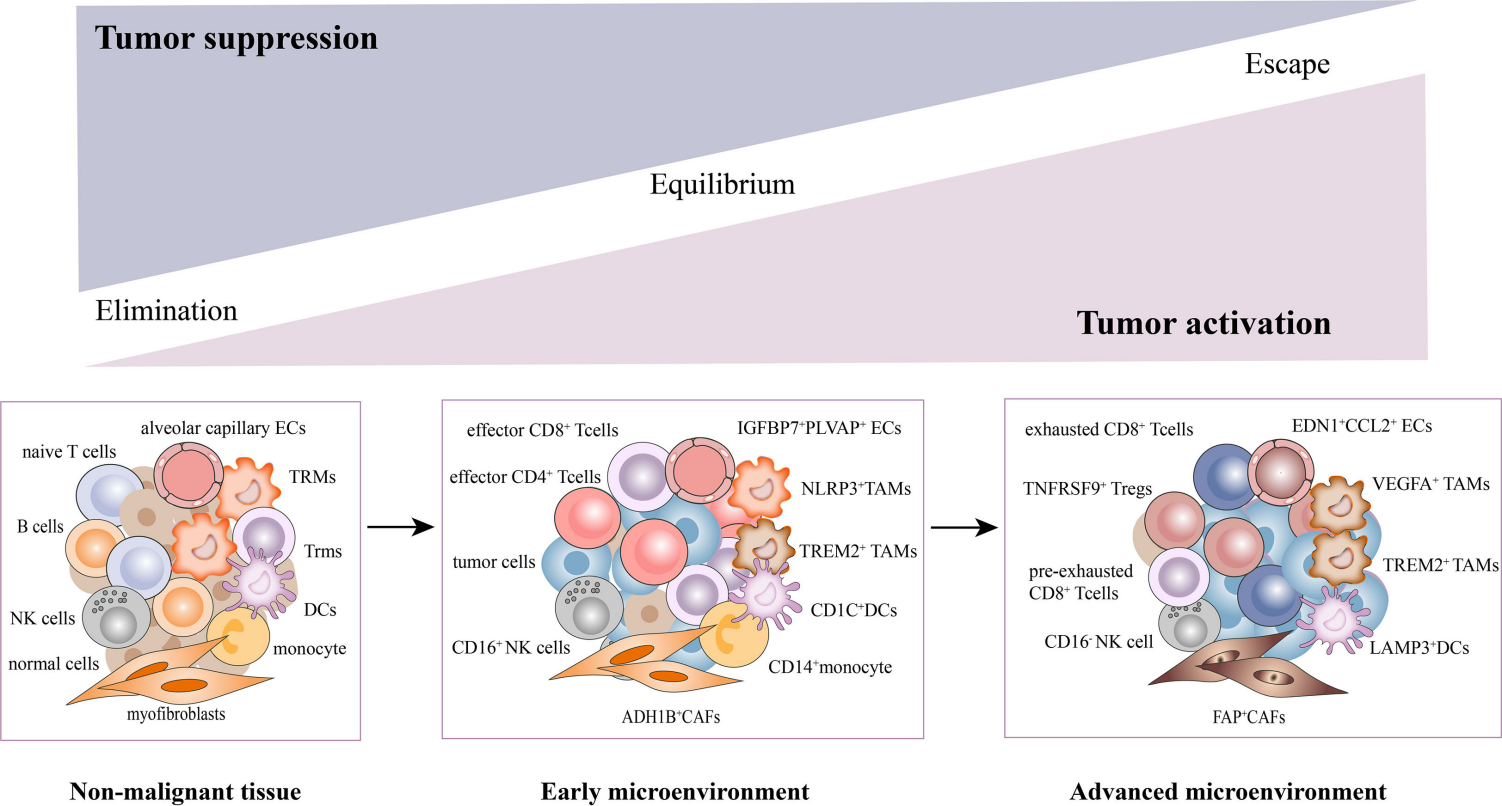
Dynamic changes of stromal subsets during tumor progression in the TME. The stromal cells undergo phenotypic remodeling and functional state shifts that drive the microenvironment toward immunosuppression, along with tumor cells. With the tumor progression, T cells underwent a gradual exhaustion process. The CD4^+^/CD8^+^ effector T cells, CD16^+^NK, NLRP3^+^TAMs, ADH1B^+^CAFs, CD1C^+^DCs, and EDN1^+^CCL2^+^ECs were mainly enriched in early tumors. While pre-exhausted, exhausted T cells, TNFRSF9^+^Tregs, CD16^-^NK, VEGFA^+^TAMs, FAP^+^CAFs (activated CAFs), LAMP3^+^DCs and IGFBP7^+^PLVAP^+^ECs were mainly found in advanced tumors.

Collectively, we have uncovered a complex cellular interplay in the TME, including the inhibition of T cell activation, recruitment of immunosuppressive cells, CAFs activation, angiogenesis, and extracellular matrix remodeling. We also elucidated the dynamic changes of stromal subsets during tumor response, thereby providing a theoretical basis for revealing novel clinical immunotherapeutic targets (**Figure 2**).

## 5 Insights into tumor immunotherapy in lung cancer

Several immunotherapies such as anti-PD-1/PD-L1 and CTLA4 have recently achieved clinical benefits (102, 103, 112–114). However, the problems of immune nonresponse and poor treatment motivate the search for novel targets and combined therapy strategies. Traditional biomarker research is mainly based on the individual level rather than specific cell subsets, resulting in limited indication value for immunotherapy. The high-resolution features of scRNA-seq can compensate for this deficiency by more accurately characterizing the heterogeneity of cell subsets in the TME to identify specific markers or cell subsets associated with associated with immunotherapy and patient prognosis (5, 23, 27, 115–117).

### 5.1 Immunological implications of tumor-infiltrating T cells

In recent years, extensive research has focused on the most promising immune checkpoints, such as LAG-3, TIM-3, and TIGIT (113, 118–121). However, the emergence of scRNA-seq technology has revealed new markers on TILs and identified new therapeutic targets. For instance, CXCR5/TCF1^+^CD8^+^ T cells have been associated with durable responses to ICB therapy in lung cancer, while LAYN^+^CD8^+^ T cells have been found to be exhausted and contributed to non-response to immunotherapy. Moreover, thymocyte selection-associated high mobility group box protein (TOX), which promotes exhaustion of tumor-infiltrating CD8^+^ T cells within the tumor, can be used to stratify patients during anti-tumor therapy, including anti-PD-1 immunotherapy (122). The TCF-1^+^CD8^+^ Texp subset has also been shown to be significantly more abundant in immune responders than non-responders (5, 117, 123–125), making TCF-1 a promising biomarker for predicting the efficacy of ICB therapy. However, clinical studies on patients are required to confirm this. Finally, the newly discovered subset of TdLN-TTSM cells has been identified as key responsive cells for PD-1/PD-L1 immune checkpoint therapy, which will become a hot topic in future tumor immunology research (24). Additionally, GNLY^+^CD4^+^ T cells are believed to be an active subset of effector CD4^+^T cells that contribute to the immune response, while TNFRSF9^+^ Tregs, a group of activated Tregs, are extensively studied for their important immunosuppressive functions. The proportion of these two cell populations was found to be highly correlated with the response to ICB therapy, indicating some new potential targets. Additionally, IL1R2, highly expressed in TNFRSF9^+^ Tregs, was associated with poor prognosis in lung cancer, making it a potential therapeutic target worth investigating (14, 126).

### 5.2 Immunological implications of myeloid cells

As the most important component of myeloid cells, TAMs have become a hot topic in the field of immunotherapy. To date, scRNA studies have revealed several new targets in lung cancer and brought new treatment options to immunotherapy such as TREM2^+^, SPP1^+^ and C1Q^+^ TAMs, which are associated with the malignant progression of lung cancer. Blocking TREM2 has shown promising anti-tumor effects in tumor patients and mouse models (20, 127) by reprogramming tumorigenic macrophages into anti-tumor macrophages, improving their impaired antigen presentation ability and inhibiting tumor growth (62, 128). Furthermore, elevated levels of SPP1 and C1Q expression are associated with a worse clinical outcome in multiple cancer types, making them potential targets for TAMs-targeting treatment (26). The NLRP3^+^ TAMs are enriched in non-cancerous tissues, which may represent a pro-inflammatory TRMs cluster and may be associated with better prognosis (48).

ScRNA-seq have revealed that LAMP3^+^ DCs may regulate the function and infiltration of specific T cell subpopulations by co-stimulatory/inhibitory ligands (26), thus exerting immunosuppressive functions. Therefore, LAMP3^+^ DCs have become a widely studied and promising target. Besides, the expansion of CD141^+^ DCs within tumors was associated with T cell activation (32), providing a crucial strategy for inducing effective anti-tumor immunity as well. In summary, combining therapies targeting immune checkpoints to achieve synergistic anti-tumor responses is the direction of immunotherapy in the future. However, further clinical studies are needed to validate these findings and assess their efficacy in patients.

### 5.3 Immunological implications of other stromal cells

In addition to targeting the subpopulations of tumor-infiltrating T cells and myeloid cells, exploring the therapeutic potential of CAFs and ECs involved in the remodeling of the TME is of great interest. In particular, ADH1B^+^ CAFs and FAP^+^ CAFs exhibit distinct immunomodulatory characteristics in the TME. Promoting the differentiation of CAFs into the ADH1B^+^CAFs may contribute to enhancing anti-tumor immune response, while FAP^+^CAFs are specifically enriched in late-stage tumors and promote tumor growth (87). The distribution of these two subtypes is associated with the prognosis of lung cancer and provides evidence for targeted therapy. Moreover, during the progression of lung cancer, the proportion of EDN1^+^CCL2^+^cECs decreases, while the proportion of IGFBP7^+^PLVAP^+^ECs significantly increases. The reshaping of EC cells into tumor ECs contributes to immune tolerance, highlighting the potential of these EC subgroups as predictive targets for immunotherapeutic responses.

### 5.4 Immunological implications of cellular modules

**Figure 3:**
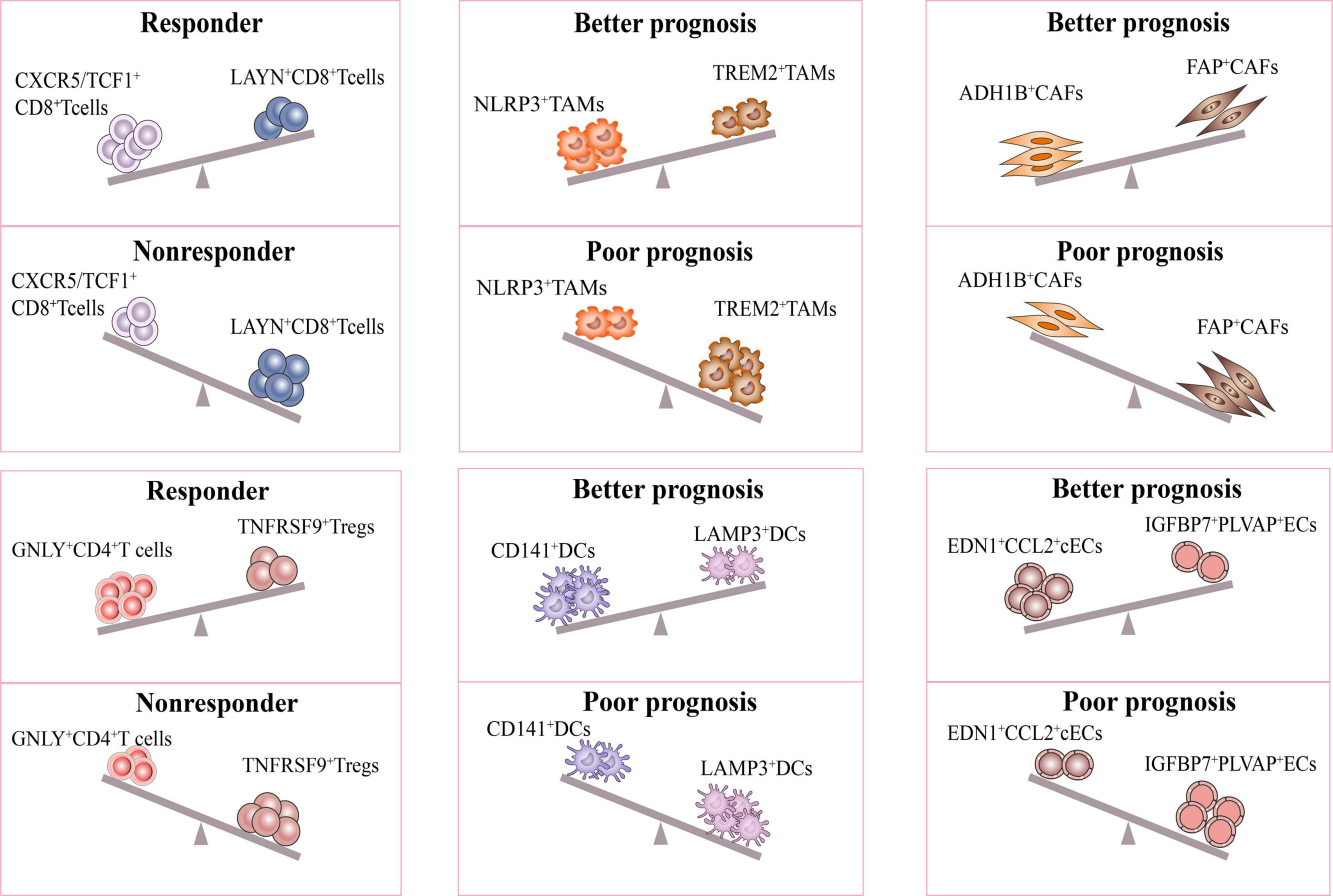
Response of immunotherapy treatment of lung cancer for distinct subsets. The figure describes influence of the distinct stromal cell phenotypes in TME on responsiveness to immunotherapy and therapeutic efficacy of treatment. The CXCR5/TCF-1^+^CD8^+^ T and GNLY^+^CD4^+^ T subsets were significantly more abundant in immune responders than in non-responders. Moreover, the ratio of NLRP3^+^/TREM2^+^ TAMs, CD141^+^/LAMP3^+^ DCs, ADH1B^+^/FAP^+^CAFs, EDN1^+^CCL2^+^ cECs/IGFBP7^+^PLVAP^+^ ECs were positively correlated with better prognosis in lung cancer. All the above subsets may be potential targets for lung cancer treatment.

It is interesting to note that recent studies have proposed models consisting of multiple cell types in the TME to predict the response and prognosis of immunotherapy. Firstly, scRNA-seq study described a cellular module termed Lung Cancer Activation Module (LCAM), which was composed of PDCD1^+^CXCL13^+^ activated T cells, IgG^+^ plasma cells, and SPP1^+^ macrophages (18). It was reported that LCAM enrichment enhances the immunotherapeutic response in NSCLC. Another study has also defined an immune-activated microenvironment called CP²E, which is composed of cancer-associated fibroblasts, macrophages derived from pro-inflammatory monocytes, plasma dendritic cells, and exhausted CD8^+^ T cells, which is associated with poor prognosis in lung cancer (129). These findings highlight the importance of considering multiple cell types in the TME when developing immunotherapies and suggest potential targets for future research (**Figure 3**).

## 6 Concluding remarks

In conclusion, this review illustrates the complex biological picture of lung cancer and illuminates the dynamics of cellular and molecular networks during tumor development. To aid future research into treatment methods, we propose novel potential targets. Combination therapy targeting inhibitory molecules and blocking specific pathways may restore innate immunity, reshape the immune microenvironment, enhance the immune response and the therapeutic efficacy of immunotherapy (32).

However, there are still some issues that need to be resolved. First, there is no uniform naming principle for cell subsets with the same function by different researchers owing to differences in methods and tissue types. Therefore, a relatively standard naming norm is needed in the future. Second, although scRNA-seq faithfully replicates global gene expression profiles, it is uncertain whether the expression levels of the transcripts truly reflect the functions of the subpopulation. Moreover, the interactions of stromal cells predicted based on ligand and receptor gene expression also need to be further verified. Hence, combining proteomics and spatial transcriptomics technologies is necessary to jointly study the functions of different subsets and cell-cell interactions in the TME. Third, on account of tumor heterogeneity, including lung cancer types (lung squamous cell carcinoma and lung adenocarcinoma), tumor sites (primary and metastatic tumors), and different populations, the classification and functions of cell subsets in the TME are heterogeneous. Therefore, finding critical subpopulations with similar functions and specific targets will assist in achieving individualized precision immunotherapy for lung cancer. Finally, due to their crucial role in immune checkpoint therapy, most current studies have focused on T cells, with limited attention given to other stromal cells in TME. In addition, although the researches on T cells are intensive, it remains to be explored whether targeting specific subsets can produce immune responses in a wide range of patients or whether immunotherapy resistance will occur during the treatment. ScRNA-seq technology is expected to be applied to explore the changes in the TME after immunotherapy and to reveal in-depth mechanisms of drug resistance. Despite these challenges, we believe that immune molecular typing based on the TME can better identify immune-responsive populations and provide a basis for precise lung cancer treatment.

## Author contributions

QZ conceived the idea, designed, and supervised the topic. QX, WP and SZ drafted the manuscript and designed the figures and tables. XW, QX, LY, ZW, XX and PZ revised the manuscript. All authors contributed to the article and approved the submitted version.
